# A simple method for determining ligament stiffness during total knee arthroplasty *in vivo*

**DOI:** 10.1038/s41598-019-41732-x

**Published:** 2019-03-27

**Authors:** Florian Völlner, Tim Weber, Markus Weber, Tobias Renkawitz, Sebastian Dendorfer, Joachim Grifka, Benjamin Craiovan

**Affiliations:** 1 Department of Orthopaedic Surgery, Regensburg University Medical Centre, Asklepios Klinikum Bad Abbach, Kaiser-Karl-V-Allee 3, 93077 Bad Abbach, Germany; 2 Department of Mechanical Engineering, OTH Regensburg, Galgenbergstraße 30, 93053 Regensburg, Germany

## Abstract

A key requirement in both native knee joints and total knee arthroplasty is a stable capsular ligament complex. However, knee stability is highly individual and ranges from clinically loose to tight. So far, hardly any *in vivo* data on the intrinsic mechanical of the knee are available. This study investigated if stiffness of the native ligament complex may be determined *in vivo* using a standard knee balancer. Measurements were obtained with a commercially available knee balancer, which was initially calibrated *in vitro*. 5 patients underwent reconstruction of the force-displacement curves of the ligament complex. Stiffness of the medial and lateral compartments were calculated to measure the stability of the capsular ligament complex. All force-displacement curves consisted of a non-linear section at the beginning and of a linear section from about 80 N onwards. The medial compartment showed values of 28.4 ± 1.2 N/mm for minimum stiffness and of 39.9 ± 1.1 N/mm for maximum stiffness; the respective values for the lateral compartment were 19.9 ± 0.9 N/mm and 46.6 ± 0.8 N/mm. A commercially available knee balancer may be calibrated for measuring stiffness of knee ligament complex *in vivo*, which may contribute to a better understanding of the intrinsic mechanical behaviour of knee joints.

## Introduction

The prerequisite for both native knee joints and total knee arthroplasty is stable ligament guidance over the full range of motion together with optimal kinematics of the knee joints. In contrast to hip joints, knee joints consist of two incongruous joint components, which must be guided over the ligaments and musculature over the full range of motion^1^. Failure to do so may result in instability, increasing leg malalignment, pain and, in the case of artificial knee joints, loosening of the components.

The natural elasticity of ligaments is highly variable, ranging from clinically loose to tight knee joints. Hyperextended knees are considered loose, whereas knees with fixed flexion deformity tend to be tight. Besides the natural elasticity leg deformities of the knee may influence stiffness of the ligaments^1–4^. For example, medial ligaments in varus knee joints seem to be tight, and lateral ligaments appear to be soft, a situation that is reversed in valgus knees. Furthermore, there are indications in the literature that the stability of ligaments is influenced by age, the sex and diseases, e.g. diabetes mellitus or rheumatoid arthritis^5–8^. Thus, Schleifenbaum *et al*. demonstrated that the tensile properties are largely variable and age-dependent in a collective aged 14–93a^5^. Chandrashekar *et al*. on the other side showed *in vitro* tests that female anterior cruciate ligament has lower mechanical properties (8.3% lower strain at failure; 14.3% lower stress at failure, 9.43% lower strain energy density at failure, and most importantly, 22.49% lower modulus of elasticity) when compared to males^9^. Breault-Janicki *et al*. could prove, that the rheumatoid tendons had higher extensibility at low stresses, lower stiffness in the linear portion of the stress-strain curve, greater rates of stress relaxation, and lower ultimate strengths than did the non-rheumatoid tendons^6^.

At the knee joint, different *in vitro* works are known, which examined single ligaments such as the medial or lateral collateral ligament or the anterior cruciate ligament^10–13^. In these studies, however, the ligaments are all investigated for their main fibre direction, a condition that is not found in the natural knee joint. In addition, the force transmission at the natural knee joint occur not by a single ligament, but by the entire knee ligament complex. Depending on the position of the knee joint different ligaments and proportions of the capsule and muscles are involved.

The aim of this study was to assess a common knee balancer currently used in orthopaedic surgery. This balancer enables the simultaneous *in vivo* measurement of tibial-femoral forces and gaps during total knee arthroplasty. These two parameters allow the reconstruction of the force-displacement curve of the medial and lateral knee ligament complex and the calculation of stiffness. By determining the stiffness, the native stability of the knee, the influence of age, diseases or axial malalignments can be investigated in large numbers directly *in vivo*. In addition, the knowledge of stiffness of the ligament complex can be used for finite element models, but also for virtual knee joints.

## Methods

### Knee balancer

**Figure 1:**
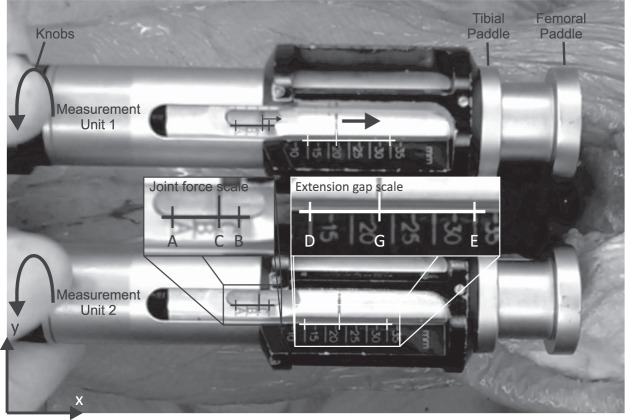
Knee balancer *in situ*. The knee balancer consists of two measuring units for the medial and lateral compartments of the knee. Paddles advanced by turning knobs clockwise. If the femoral paddle has contact with the femur, more and more force is applied. The current force and extension gap can be read at the joint force scale resp. extension gap scale at each of the two measuring units. For reconstruction of the force-displacement curve, we determined the coordinates (origin left bottom corner x, y) of A, B and C for the force scale and D, E and G for the extension gap at different time points during extension separated for each measuring unit.

The ‘knee balancer’ developed by P.F.C Sigma and LCS Complete EGF Instrumentation of Depuy (Depuy Synthes, Warsaw, USA) was used for determining and reconstructing the force-displacement curve of the medial and lateral ligament complex (Fig. 1). This knee balancer consists of two measuring units for the medial and lateral compartments of the knee. Each unit has a rigid tibial paddle and a mobile femoral paddle, which are connected to a knob with a spring. The femoral paddle is extended by turning the knobs clockwise, the axial feed can be read at the extension gap scale (Fig. 1). If the femoral paddle has contact with the condyles, force is applied via a spring to the femur. The current force can be read at the joint force scale (Fig. 1).

### Experimental measurements

**Figure 2:**
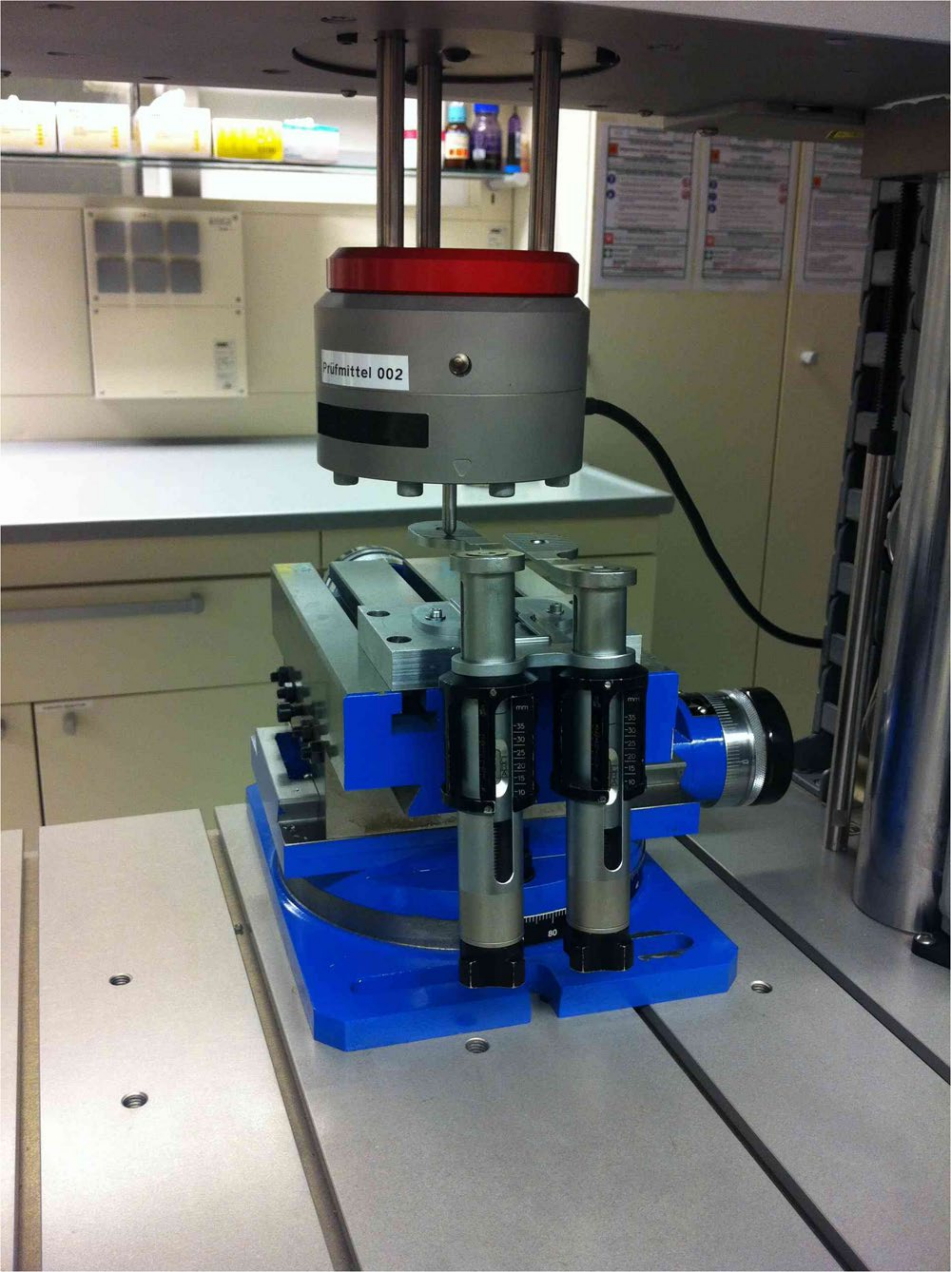
For the experimental measurements, the knee balancer was mounted on a sliding table and placed in an electromechanical universal testing machine (ElectroPuls E3000 Instron, Load cell Dynacell Series 2527, High Wycombe, UK). With this experimental setup the reliability, validity and the linearity could be proven.

**Figure 3:**
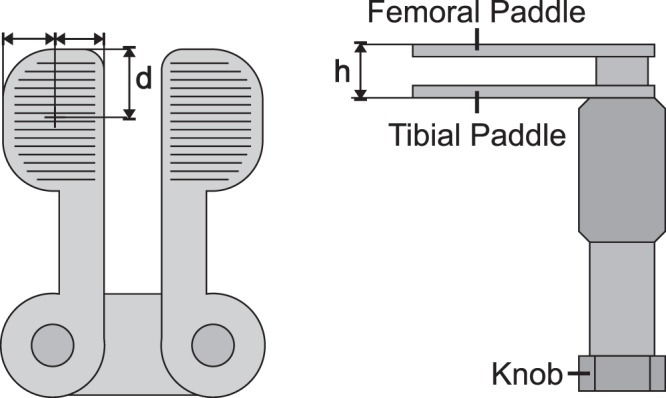
Scheme of the knee balancer. To show reliability, applied forces were determined at different distances d (d = 10 and 30 mm, measured from the front edge) and at different heights h (h = 10, 20 and 40 mm) by a universal testing machine.

**Figure 4:**
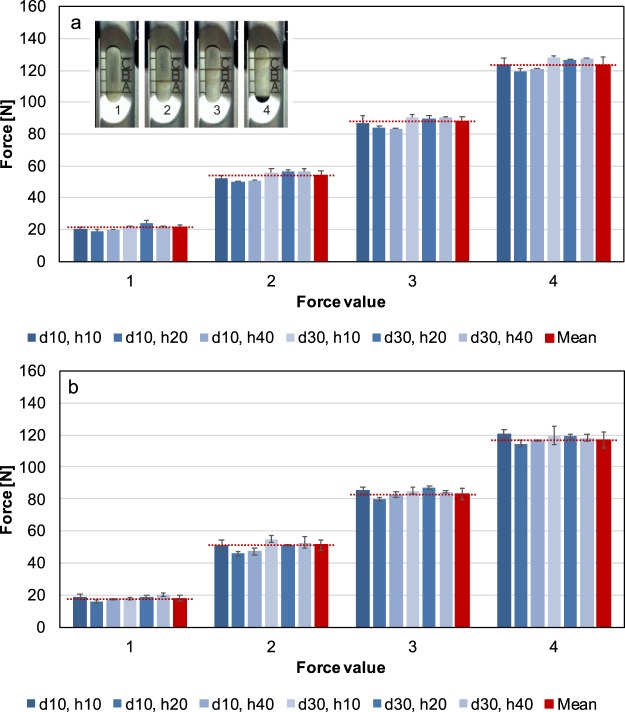
Applied forces on the knee balancer for left (**a**) and right (**b**) measuring unit determined by the static testing machine at measurement points 1–4 at joint force scale (corresponding to the lines of the joint force scale, please refer insert **a**). The forces were measured at different distances d (measurement points on the paddles: paddle centred with the distance 10 and 30 mm, measured from the front edge of the paddle) and at different heights h (10, 20 und 40 mm) of the paddle by universal testing machine. In red, the corresponding mean value is given.

Validity and Reliability of the knee balancer were shown by means of an electromechanical universal testing machine (ElectroPuls E3000 Instron, High Wycombe, UK; Fig. 2) with a dynamic load cell (2527 series Dynacell, Instron, High Wycombe, UK.). The capacity is ± 5 kN with an accuracy of ± 0.25%. The sensing rate was 100 Hz. For measurements the knee balancer was screwed with the tibia paddle onto a sliding table and the height h (h = 10, 20, 40 mm) of the paddle was adjusted (Fig. 2). Then, the balancer was placed under load cell of the Instron machine at measuring point, so that there was just a contact between the load cell and the paddle (measuring points: paddle centred with the distance d = 10 and 30 mm, measured from the front edge of the paddle, see Fig. 3). We set a defined force via the knee balancer, corresponding to the lines on the joint force scale (measurement points 1–4, small insert (Fig. 4a)). The applied forces were recorded by the testing machine (Fig. 4). Each measurement was repeated three times at each point.

**Figure 5:**
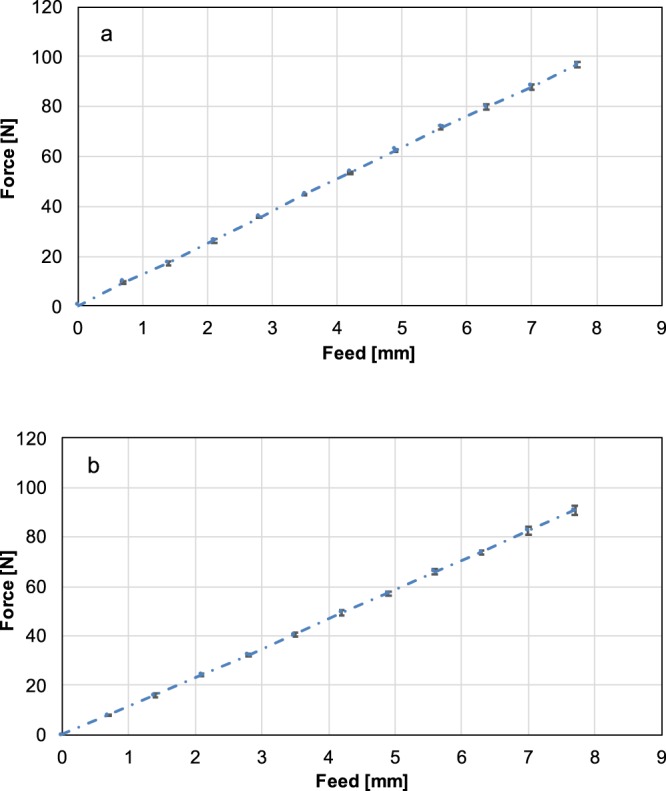
Force-feed-diagram of the left (**a**) and right (**b**) paddle. Constant feed of the knee balancer leads to constant increase in force at universal testing machine. Each measurement was repeated three times. The dash lines represent the linear fit to the feed-force data. The biomechanics of the knee balancer correspond to that of a spring system with a spring constant D = 12,0 N/mm for the left paddle and D = 11,8 N/mm for the right paddle.

To show the linearity of the knee balancer, we used the same setup as in the proof of reliability and validity. The knee balancer was also mounted on the sliding table (Fig. 2), but the femoral paddles were fully retracted. The balancer was placed with the tip of the femoral paddle under the load cell of the static testing machine (paddle centred with the distance of 10 mm, measured from the front edge of the paddle), so that the load cell had just contact with the paddle. Then force was applied by quarter turns of the knob (corresponds to a feed increment of the femoral paddle of 0.7 mm) via knee balancer. After every quarter turn the applied force was determined by the testing machine (Fig. 5). Each measurement was repeated three times at each side.

### Ethical approval and informed consent

Ethical approval was given by the Ethics Committee for research on human beings in Regensburg, Germany (Reference Number: 12-101-0228). The study was carried out in accordance with the ethical standards of the Declaration of Helsinki of 1975. Patients were informed in written form as well as orally by the study personnel. An informed consent was obtained from all subjects. Participation was voluntary, and withdrawal was possible at any time.

### Patient data

After approval by the local Ethics Committee the force-displacement curves of 5 patients (3 men and 2 women) were reconstructed (Table 1). The average age of the patients at surgery was 72 ± 10 a, and the mean BMI was 30.6 ± 4.5 kg/m^2^. The leg axis was on average 176.1 ± 5.4°. All patients underwent surgery because of primary osteoarthritis grade 3 to 4 classified according to Kellgren and Lawrence. Exclusion criteria were secondary arthritis, post-traumatic deformities, injuries and previous knee surgery.

**Table 1:** Summary of patient characteristics and stiffness of the medial ligament complex (MLC) and the lateral ligament complex (LLC).

Specimen	Sex	Age [a]	BMI [kg/m^2^]	Leg side	Leg axis [°]	Stiffness of MLC Mean (SD) [N/mm]	Stiffness of LLC Mean (SD) [N/mm]
1	M	72	31.9	left	176.5	34.6 (1.1)	46.6 (0.8)
2	F	82	33.3	right	175.5	28.4 (1.2)	27.0 (1.0)
3	M	58	36.6	left	177.3	29.4 (0.6)	19.9 (0.9)
4	M	63	27.4	left	167.2	28.7 (1.0)	27.5 (1.5)
5	F	85	23.7	right	184.0	39.9 (1.1)	33.1 (0.2)

### Surgical technique and *in vivo* measurements

Force-displacement curves were determined according to our standard surgical routine for total knee arthroplasty^14,15^. First, a mid-line skin incision was made, and the capsule was opened according to the medial parapatellar approach, followed by the resection of the anterior cruciate ligament and the menisci. Two Schanz screws were bicortically drilled into the femur and tibial plateau outside the joint capsule to avoid soft tissue damage. Subsequently, the passive optical reference arrays were fixed (Brainlab AG, Munich, Germany). According to the navigation workflow, the femoral head centre was determined by circumduction. Anatomical landmarks on the tibia and femur were identified by means of a pointer (femoral: distal femoral knee centre, medial and lateral epicondyle, Whiteside line, articulating surface of the medial and lateral condyle; tibial: tibial plateau size, medial and lateral malleolus, Akagi line as tibial AP axis and the articulating surface of the medial and lateral tibial plateau). Leg alignments were recorded in full extension and in 90 degrees flexion. According to our standard surgical routine, the tibial slope was set to 4 degrees posterior slope, and the tibial cutting jig was aligned according to the recommendation of the navigation system perpendicular to the mechanical axis of the tibia. The jig was fixed, and the tibial cut was made, removing 8 mm of bone and cartilage of the healthy compartment. During all preparation steps, the collateral ligaments were thereby protected by retractors. A release of the ligaments was not performed. After removal of the jig, the cut was verified, and the knee balancer was placed into the extension gap between tibia and femur. The leg was straightened, and a preload of about 10 N to 20 N was applied to the medial and lateral ligamentous complex. The knee ligament complex was extended manually up to the maximum possible force of 170 N, resulting in a strain rate from approx. 0.4 to 0.6% s^−1^ depending on the speed of extension and the length of the ligaments. These rates are considered to be quasi-static, so that viscoelastic effects are of no importance^16^. Expansion was recorded on video (Canon Legria HF R506 Full HD Camcorder, frame rate 25 s^−1^, Tokyo, Japan) and evaluated after surgery. Each measurement was repeated twice for each compartment (medial and lateral).

### Reconstruction of the force-displacement curve

For reconstructing the force-displacement curve, the videos were imported into Matlab (R2013a, Mathworks, Natick, USA). Every 10th frame (approx. 2 frames per second) was analysed, so that 15–20 points were evaluated for each reconstruction. In every frame the coordinates (origin left bottom corner, x, y) of 12 points – 6 points per unit (joint force scale: A, B, C; extension gap scale: D, E, G) – were determined (Fig. 1).

The coordinates of these points were imported into Excel (Version 16, Microsoft Corp. Redmond, USA). In a first step, the distances AB and AC were calculated:

$$\overline{AB}=\sqrt{{({A}_{x}-{B}_{x})}^{2}+{({A}_{y}-{B}_{y})}^{2}}$$

$$\overline{AC}=\sqrt{{({A}_{x}-{C}_{x})}^{2}+{({A}_{y}-{C}_{y})}^{2}}$$

The distance AB corresponds to the reference range of the scale, while AC represents the distance of the measuring point C from the scale start point A. The relativity factor m describes the proportion of the distance AC to the reference distance AB:

$$m=\frac{\overline{AC}}{\overline{AB}}$$

The current force can be calculated according to the measured forces F_A_ and F_B_ at the endpoints A and B (corresponding to the applied mean forces at measurement point 1 and 4 of the laboratory tests (Fig. 4); left paddle: F_A_ = 21.2 N, F_B_ = 123.3 N; right paddle: F_A_ = 18.1 N, F_B_ = 116.9 N) and the relativity factor m:

1 $${F}_{C}={F}_{A}+{m}\ast ({F}_{B}-{F}_{A})\,\,[N]$$

Displacement was calculated in a similar manner. First, the distances DE and EG were determined from the coordinates of the points D, E, G (Fig. 1):

$$\overline{DE}=\sqrt{{({D}_{x}-{E}_{x})}^{2}+{({D}_{y}-{E}_{y})}^{2}}$$

$$\overline{EG}=\sqrt{{({E}_{x}-{G}_{x})}^{2}+{({E}_{y}-{G}_{y})}^{2}}$$

In reality, the distance DE corresponds to a length of 20 mm according to the scale and is used to calibrate the individual frames in order to calculate tilting or magnification errors by the camera position (Fig. 1). Via cross multiplication, the real distance EG_real_ can be calculated in mm:

$$E{G}_{real}=20\,{mm}\ast \frac{\overline{EG}}{\overline{DE}}\,[mm]$$

Displacement p corresponds to the change in the distance EG_real_ compared to frame 1 and can be calculated as follows:

2 $$p=E{G}_{real,frame1}-E{G}_{real,currentframe}$$

**Figure 6:**
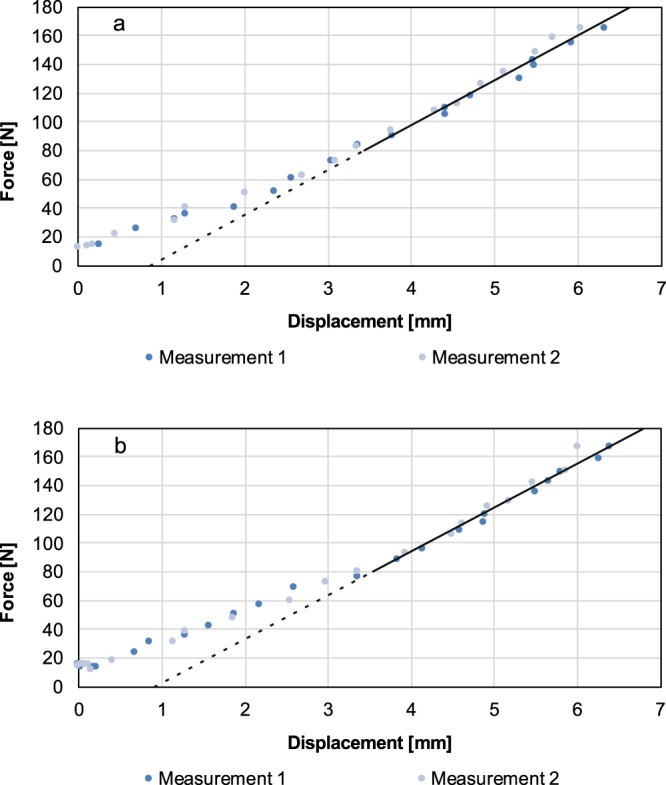
*In vivo* determination of the stiffness of the medial (**a**) and lateral (**b**) compartment. The graph presents the force as a function of the displacement. Values for stiffness of the medial and lateral compartment were derived from the gradient of the linear section of the curve. The transition to linearity is individual, considering all measurements, a linearity is certainly given from 80 N. The steeper the gradient line, the stiffer are the ligament complex.

The force-displacement curve (Fig. 6) is obtained by plotting force F_C_ (Eq. 1) against displacement p (Eq. 2).

## Results

### Experimental measurements

The results of force measured at different positions of the paddles (measurement point 1–4) are presented in Fig. 4. For the left paddle, the mean forces for the knee balancer are 21.2 ± 1.8 N at measurement point 1, 53.6 ± 3.3 N at measurement point 2, 87.6 ± 3.5 N at measurement point 3 and 123.3 ± 5.0 N at measurement point 4. For the right paddle, the values are 18.1 ± 1.8 N (1), 51.3 ± 3.5 N (2), 83.4 ± 3.6 N (3) and 116.9 ± 4.8 N (4). Due to the fact that differences are found between the two measuring units, it must be taken into account in the *in vivo* measurements which measuring unit was used for the lateral respectively for medial compartment.

The force-feed diagram for the left and right paddle is shown in Fig. 5.

Constant feed leads to constant increase in force, which corresponds to a simple spring system with a spring constant of 12.0 ± 1.2 N/mm for the left paddle and of 11.8 ± 1.8 N/mm for the right paddle.

### *In vivo* measurements

Figure 6 shows force as a function of displacement for the medial and lateral compartments. Results of the *in vitro* experiments are summarised in Table 1. The curve can be divided into different sections. The first section is non-linear with low initial stiffness and corresponds to the extension of the crimped collagen fibres^17^. The second section is linear. The slope of this linear section of the force-displacement curve is defined as stiffness of the ligament complex representing the structural properties. The transition to linearity is fluent. Considering all measurements, the linearity is certainly given from an applied force of 80 N. The steeper the linear section, the stiffer are the ligaments. For the medial compartment, mean values are 28.4 ± 1.2 N/mm for minimum stiffness and 39.9 ± 1.1 N/mm for maximum stiffness; for the lateral compartment, the respective values are 19.9 ± 0.9 N/mm and 46.6 ± 0.8 N/mm.

## Discussion

In the present study, a standard ligament balancer was calibrated for the *in vivo* determination of stiffness of the medial and lateral ligament complex during total knee arthroplasty. Linearity, validity and reliability measurements were demonstrated in laboratory tests. Constant linear spreading via a femoral paddle leads to linear increase in force in the static testing machine. Furthermore, the measurement of force is independent of the position of the femoral paddle. The reconstructed force-displacement curves *in vivo* are composed of a non-linear part followed by a linear part. This characteristic corresponds to the behaviour known from the structural properties of bone ligament bone complexes, for instance of separate knee ligaments or Achilles tendons *in vitro*^11,18^. Apart from a work of our group on the stiffness of patients *in vivo* compared with the Thiel-embalmed cadaver, there are no data on the stiffness of the entire knee ligament complex^15^. Our vivo measurements yielded mean stiffness values of 32.2 ± 4.5 N/mm for the medial ligament complex and 30.9 ± 8.9 N/mm for the lateral ligament complex. In the literature, stiffness has only been described for isolated ligaments *in vitro*^7,12,13,18,19^. The authors are not aware of any stiffness measurements of entire medial and lateral compartments. So, Sugita *et al*.^20^, for instance, described a linear stiffness value of 58.1 ± 22.8 N/mm for the lateral collateral ligament in 10 cadaveric knees. When examining the medial collateral ligament complex of cadaveric knees, Robinson *et al*.^19^ found a stiffness value of 80.0 ± 8.0 N/mm for the superficial ligament complex and 42.0 ± 14.0 N/mm for the deep medial collateral ligament complex − findings that were higher than the values found in this study. The reason for this discrepancy is probably that, *in vivo*, all ligamentous structures are oblique to the direction of extension in the straightened leg in contrast to *in vitro* measurements. Thus, the superficial medial collateral ligament is relaxed in straightened legs, most effectively at 60 degrees of flexion and less effective at full extension, whereas the deep medial collateral ligament is tighter in extension^21^. On the lateral side, lateral collateral ligaments are tilted posteriorly in extension when passing from the femur to the fibula^20^. The oblique force application results in lower stiffness values. But larger study populations are required to obtain a validated statement about knee stability. Furthermore, it should be noted that only passive stabilisers such as ligaments and capsule portions were recorded in our study. No active stabilisation by the knee joint musculature was detected, a factor that we estimate to be very high *in vivo*.

The current study has some limitations. Static calibration and shifts imposed by mechanical friction were caused by the construction of the ligament balancer. Second, stiffness could be readily determined in this small section of the linear part of the force-displacement curve until 170 N. In various cadaver studies, single ligaments were extended up to the ultimate tensile strength of 600 N, enabling stiffness calculations over a larger linear section of the force-displacement curve^19,20,22^. To improve accuracy, however, it would be helpful to be able to determine stiffness not only in a linear but also in a larger range. Higher forces by means of the knee balancer may injure the capsular ligaments and is thus not feasible.

Ligament balancing, which is an important factor for good function and survival after total knee arthroplasty, necessitates the quantification of the tibial-femoral force in the medial and lateral ligaments over the full range of motion. In this study, stiffness was measured only at the extended leg. Assessing overall knee stability requires measurements over the full range of motion. The aim of this study was to show that determination of stiffness with a common ligament balancer is possible. Only then can the examination of the knee joint stability be carried out in large numbers and putative mechanical or demographic influencing factors such as the leg axis, age or gender or pre-existing diseases are examined.

## Conclusions

In the present study, we were able to validate a common ligament balancer as a measuring instrument. Furthermore, initial *in vivo* stiffness determinations could be performed. The quantification of such a parameter can provide useful *in vivo* information for a better understanding of the complex knee joint, its influencing factors such as leg axis, age, gender or release techniques and thus can help validate the way surgeons implant knee prosthesis.
